# Clinical, Biochemical, and Electrocardiographic Predictors of Mortality in Acute Aluminum Phosphide Poisoning

**DOI:** 10.1155/jt/4876498

**Published:** 2026-09-22

**Authors:** Raghda H. Deraz, Omar Saafan, Laila M. E. Sabik, Shymaa M. N. Abdulrhman

**Affiliations:** ^1^ Department of Forensic Medicine and Clinical Toxicology, Faculty of Medicine, Zagazig University, Zagazig, Egypt, zu.edu.eg; ^2^ Department of Respiratory Medicine, Faculty of Medicine, Helwan University, Cairo, Egypt, helwan.edu.eg

**Keywords:** aluminum phosphide, arterial blood gas, cortisol, electrocardiographic, poisoning, prognostic markers, renin, Simplified Acute Physiology Score II (SAPS II), troponin I

## Abstract

**Background:**

Aluminum phosphide (AlP) poisoning is among the deadliest pesticide poisonings, particularly in developing agricultural communities, with multiorgan involvement significantly affecting the poisoned patient’s outcome.

**Objective:**

To evaluate the prognostic significance of arterial blood gas (ABG), serum troponin I, cortisol, renin, early ECG abnormalities, and the Simplified Acute Physiology Score II (SAPS II) in predicting the outcome of acute AlP poisoning during the first 24 h postingestion.

**Methods:**

A prospective cohort study was conducted on 42 patients with confirmed acute AlP ingestion who met the inclusion criteria at Zagazig University Hospitals. All patients underwent thorough history‐taking, clinical assessment, routine laboratory investigations, and selected specific tests, including ECG assessment, measurement of serum troponin I, cortisol, and renin, ABG analysis, and finally SAPS II scoring. Patients were categorized as survivors or nonsurvivors. The predictive ability of several parameters for mortality was assessed using the *t*‐test and ROC curve analysis.

**Results:**

Mortality rate was 59.5%. Abnormal ECGs were significantly more common in nonsurvivors (*p* < 0.001). Renin (AUC = 0.81) and cortisol (AUC = 0.69) demonstrated fair‐to‐good predictive accuracy, whereas troponin I showed poor prognostic value (AUC = 0.48). SAPS II demonstrated very high discriminatory performance (AUC = 1.00). SAPS II also correlated positively with serum cortisol and renin, and negatively with pH, bicarbonate, and base excess.

**Conclusion:**

SAPS II had the highest validity as a mortality predictor for AlP poisoning, followed by bicarbonate, base excess, renin, pH, and cortisol, arranged in descending order according to their AUC. ECG evaluation is also a reliable early predictor of mortality. These parameters can aid early risk stratification and guide timely intensive management.

## 1. Introduction

Aluminum phosphide (AlP), commonly referred to as the “wheat pill,” is a solid fumigant widely used to preserve grains against pests. Due to its low cost, high efficacy, and unrestricted availability, it is frequently used in developing countries such as Egypt, India, and Iran [1]. However, its toxicity is profound. Once ingested, AlP reacts with water and hydrochloric acid in the stomach to release phosphine gas (PH_3_), a potent mitochondrial poison that leads to multiorgan dysfunction and rapid death [2, 3].

Patients typically present with a range of nonspecific yet severe symptoms, including gastrointestinal irritation, hypotension, arrhythmias, metabolic acidosis, and shock. The case fatality rate of AlP poisoning is alarmingly high, ranging from 30% to over 70% across studies, often because of delayed presentation, lack of specific antidotes, and rapid systemic deterioration [3, 4].

The cardiovascular system is among the earliest affected systems in AlP toxicity. Phosphine‐induced myocardial depression can manifest as electrocardiographic (ECG) abnormalities, including conduction blocks, ischemic changes, and fatal arrhythmias [5]. Elevated serum biomarkers such as troponin I may reflect direct myocardial injury, while endocrine stress responses—represented by elevated cortisol and renin levels—may provide additional prognostic insights [6, 7].

Prognostic scoring systems like the Simplified Acute Physiology Score II (SAPS II) combine demographic, clinical, and laboratory data to estimate disease severity and predict mortality objectively. Although SAPS II has been validated in intensive care settings, few studies have assessed its utility specifically in acute AlP poisoning [7–10].

Moreover, due to the inevitable development of metabolic acidosis due to tissue hypoxia, assessment of arterial blood gas (ABG) parameters—particularly pH, bicarbonate (HCO_3_^-^), and base excess—is a critical indicator of severely poisoned patients [6, 11–13].

Despite numerous case reports and observational studies, the literature lacks a comprehensive evaluation of multiple prognostic indicators assessed within the critical first 24 h postingestion. This study addresses that gap by assessing the prognostic performance of ABG, troponin I, cortisol, and renin levels, ECG findings, and SAPS II score in a cohort of patients with confirmed acute AlP poisoning.

## 2. Subjects and Methods

### 2.1. Study Design, Population, and Ethical Considerations

This was a prospective cohort study conducted at the Poison Control Center–Zagazig University Hospitals (PCC‐ZUH), the emergency department (ED), and the intensive care units (ICU) between January 2021 and June 2021. A prior sample size estimate was performed by the Faculty Biostatistics/Research Support Unit, based on the primary endpoint of in‐hospital mortality. The minimum required sample size was 36 patients. During the study period, 42 consecutive eligible patients were enrolled.

**FIGURE 1:**
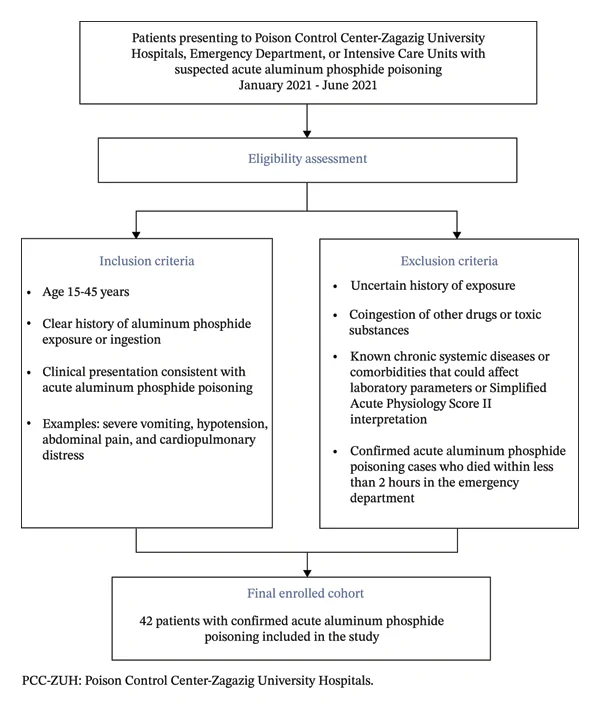
Flowchart of patient’s eligibility and study inclusion.

Inclusion criteria comprise patients aged 15–45 years, with a clear history of exposure or ingestion, clinical presentation consistent with acute AIP poisoning, e.g., severe vomiting, hypotension, abdominal pain, and cardiopulmonary distress. Cases with an uncertain history of exposure, or a history of coingestion of other drugs or toxic substances, and those with known chronic systemic diseases or comorbidities that could affect laboratory parameters and SAPS II scoring were excluded. Confirmed cases of acute AlP poisoning who died shortly in the ED (< 2 h) were excluded as well (Figure 1).

The study was performed according to the World Medical Association Declaration of Helsinki after approval from the scientific research ethical committee (Institutional Research Board [IRB] approval number ZU‐IRB #6655/13‐1‐2021). Informed written consent was obtained from the patient (if conscious and adult) or their relatives. The patients’ records were kept confidential in all stages of the study and were used for research purposes only. All cases in this study were resuscitated and received the required treatment for such cases.

### 2.2. Patient Assessment and Management

All enrolled patients underwent standardized evaluation, including

 A. History taking of demographic data (especially age and sex) and the poisoning history (the alleged consumed amount, and the time‐lapse between intake and hospital presentation).
 B. Clinical assessment of vital signs (blood pressure, heart rate, respiratory rate, and temperature) and consciousness level using the Glasgow Coma Scale (GCS) [14].
 C. Laboratory investigations: Blood samples were withdrawn at the time of admission as follows: About 1‐2 cm^3^ of arterial blood was withdrawn in a heparinized syringe to perform ABGs by an automated analyzer (Cabs B 221) according to the method described by Williams [15], and approximately 7 cm^3^ of venous blood was collected in a plain tube, allowed to clot, and then centrifuged for 10 min at 5000 rpm to collect the serum supernatant samples used for measuring biomarkers of troponin I, cortisol, and renin levels
 D. Standard twelve‐lead ECG was recorded for all cases on arrival at the university hospital ED to detect early probable abnormalities that may impact the patient prognosis. A resident cardiologist in the ED interpreted ECGs and then confirmed by a well‐trained senior toxicologist partner according to the standard criteria. The designed abnormalities to look for were (1) ischemic changes, defined as ST‐segment elevation or depression ≥ 1 mm in at least two adjacent leads, or T‐wave inversion or flattening, (2) conduction abnormalities, defined as atrioventricular block (first, second, or third degree), bundle branch block, or widened QRS complexes (> 120 msec) with subsequent prolonged QT interval, and (3) ventricular arrhythmias (ventricular tachycardia and ventricular fibrillation). ECG interpretation was not blinded to clinical status or outcome.

All cases were treated according to the management protocol adopted in PCC‐ZUH, including initial patient stabilization, decontamination with almond, coconut, or paraffin oil, and advanced supportive measures such as vasoactive agents and inotropes (e.g., noradrenaline and dobutamine), sodium bicarbonate, steroids, N‐acetylcysteine, magnesium sulfate, and calcium gluconate. All surviving patients were discharged 48 h after normalization of vital signs and laboratory findings, following consultation with a cardiologist and a psychiatrist.

### 2.3. Scoring System and Patient Classification

Following proper assessment and emergency stabilization, poisoned cases were scored within the first 24 h of admission using the SAPS II, incorporating the demographic history, clinical findings, and laboratory investigations [16]. The adopted SAPS II scoring in the current study was slightly modified to fit the acute AlP‐poisoned patients [8].

According to their outcome during the first 24 h of admission, the involved patients were classified into either survivors (complete recovery) or nonsurvivors (in‐hospital death).

### 2.4. Statistical Analysis

Data were analyzed using IBM SPSS Statistics for Windows, Version 27.0 (IBM Corp., Armonk, NY, USA). Quantitative variables were expressed as mean ± standard deviation (SD) for normally distributed data or median (interquartile range [IQR]) for nonnormally distributed data. Qualitative variables were presented as frequencies and percentages. Comparisons between two groups were performed using the independent *t*‐test for normally distributed continuous variables and the Mann–Whitney *U* test for nonnormally distributed variables. The chi‐square test was used for categorical data. Correlations between quantitative variables were assessed using Pearson’s or Spearman’s correlation coefficients as appropriate.

Receiver operating characteristic (ROC) curve analysis was conducted to evaluate the predictive performance of selected parameters and to determine optimal cutoff values. The area under the curve (AUC) was used as a measure of discriminatory ability. A *p* value < 0.05 was considered statistically significant. No formal correction for multiple comparisons was applied, as analyses were exploratory and hypothesis‐driven.

## 3. Results

### 3.1. Demographic Data, Poisoning History (Number of Tablets and Time Lapse), and the Overall Mortality

A total of 42 patients were included, with a mean age of 22.7 ± 8.9 years; 69% were female. The overall mortality rate was 59.5% (*n* = 25). There was no significant relationship between patient sex or age and the outcome. Poisoning characteristics did not significantly differ between survivors and nonsurvivors. The median ingested amount was one tablet in both groups, and the median lag time to hospital presentation was approximately 3 hours, with no statistically significant association with outcome (*p* > 0.05) (Table 1).

**TABLE 1:** Statistical comparison between survivors and nonsurvivors regarding sex, age, ingested amount, and time lapse.

Variable		Survivors (*n* = 17)	Nonsurvivors (*n* = 25)	MW/*χ* ^2^	*p*
Age, years	Median (IQR)	21 (17–25)	18 (16–25)	0.58	0.56 NS

Sex	Male Female	13 (76.5%) 4 (23.5%)	16 (64%) 9 (36%)	0.74	0.39 NS

Amount	Median (IQR)	1 (0.5–1)	1 (0.5–1)	1	0.32 NS

Time lapse (h)	Median (IQR)	3 (2–3)	3 (2–4)	0.55	0.58 NS

*Note:* IQR: interquartile range, MW: Mann–Whitney test, NS: nonsignificant (*p* > 0.05), and *χ* ^2^: chi‐square test.

### 3.2. Clinical Assessment

Clinical data, including heart rate, blood pressure, respiratory rate, temperature, oxygen saturation, and GCS, were collected from all cases and incorporated into SAPS II to score patients’ severity of poisoning.

### 3.3. ABG and Serum Biomarkers

There was a highly significant decrease in PH, HCO_3_, and BE levels in the nonsurvivors compared with the survivors (Table 2). Table 2 also demonstrates a significant rise in cortisol level and a highly significant rise in renin in the nonsurvivors, while troponin I was insignificantly different in both groups.

**TABLE 2:** Statistical comparison of laboratory parameters between survivors and nonsurvivors.

Variable		Survivors (*n* = 17)	Nonsurvivors (*n* = 25)	*t*/MW	*p*
PH	Mean ± SD	7.36 ± 0.11	7.22 ± 0.22	2.53	0.015^∗^
HCO_3_ (%)	Median (IQR)	17.6 (15.6–21)	10.2 (8.7–13.9)	4.29	< 0.001^∗∗^
BE (mEq/L)	Median (IQR)	−5.9 (−5.6/−9)	−12.4 (‐10.7/‐15.6)	3.9	< 0.001^∗∗^
Troponin I (ng/mL)	Median (IQR)	< 0.1	< 0.1	0.13	0.90 NS
Cortisol (μg/dL)	Median (IQR)	19.48 (11.31–28.48)	38.74 (14.82–45.59)	2.11	0.03^∗^
Renin (pg/mL)	Median (IQR)	43 (34–51)	135 (123–234)	3.37	0.001^∗^

*Note:* ALT: alanine transferase enzyme, AST: aspartate aminotransferase, CAT: catalase, HCO_3_: bicarbonate, IQR: interquartile range, MDA: malondialdehyde, mEq: milliequivalent, MW: Mann–Whitney test, NS: nonsignificant (*p* > 0.05), PH: the potential of hydrogen, SOD: superoxide dismutase, *t*: independent *t*‐test.

Abbreviations: BE: base excess, BUN: blood urea nitrogen, SD: standard deviation, TLC: total leukocytic count.

^∗^Significant (*p* < 0.05).

^∗∗^Highly significant (*p* < 0.001).

### 3.4. Twelve‐Lead ECG Changes

Normal ECG traces were reported in only 8 cases (19%) of all enrolled cases. Ischemic changes (ST depression/elevation and T wave abnormalities) were the most frequent abnormal ECG finding among the studied cases (24%). Other abnormalities are presented in Table 3.

**TABLE 3:** ECG findings among the aluminum phosphide‐poisoned cases.

Variable	(*n* = 42)	
	No	%
Normal (sinus rhythm)	8	19
Sinus tachycardia	5	11.9
Sinus bradycardia	2	4.8
Atrial flutter	3	7.1
Heart block	5	11.9
Ischemic changes (ST depression/elevation, T wave abnormalities)	10	24
Wide QRS/ventricular tachycardia, and/or ventricular fibrillation	9	21.4

**FIGURE 2:**
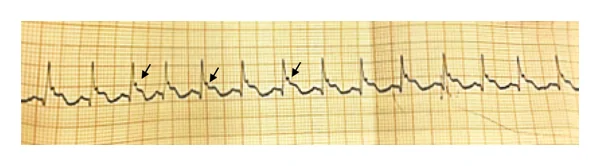
ECG trace with ischemic changes (elevated ST segment and Osborn wave “arrows” suggestive of bundle branch block).

**FIGURE 3:**
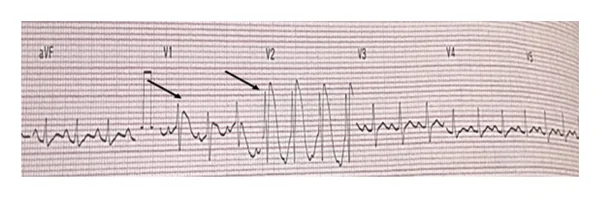
ECG trace with right ventricular ischemic changes and right bundle branch block (QRS > 120 ms, elevated ST segment with RSR⸌ pattern with Brugada pattern type a (elevated ST segment with inverted T wave) in the anterior chest leads V1, V2 “M‐shaped QRS,” and wide slurred S wave in lateral leads V4‐5).

**FIGURE 4:**
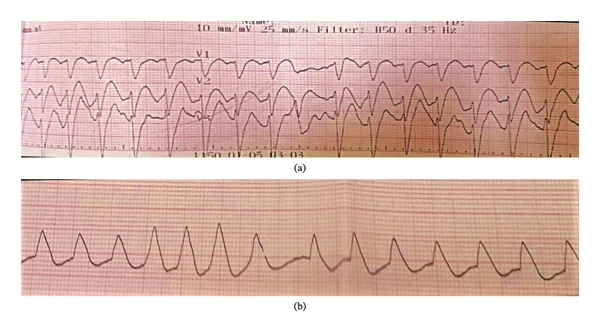
(a and b) Ventricular tachycardia (wide, bizarre QRS complex, HR > 200 bpm).

**FIGURE 5:**
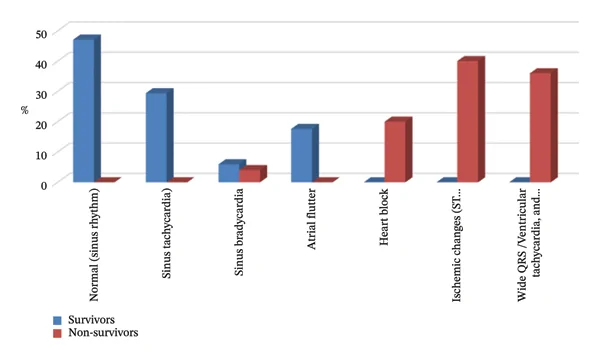
Comparison between survivors and nonsurvivors in ECG findings.

There was a statistically significant increase in the frequency of ischemic changes (40%) (Figures 2 and 3), ventricular dysrhythmia (wide QRS/ventricular tachycardia (Figure 4a,b) and/or V. fibrillation), representing 36%, and heart block (20%) among nonsurvivors compared with the survivors (*p* value < 0.001). 47.1% of survivors showed normal ECG patterns (Figure 5).

### 3.5. SAPS II Score and Predicted Mortality

The severity of illness was assessed using the SAPS II score, which incorporates systolic blood pressure, heart rate, oxygenation, renal function, and other physiological parameters. There was a highly significant increase in the SAPSII score and its ability to predict mortality among nonsurvivors compared with survivors (*p* < 0.001) (Table 4).

**TABLE 4:** Statistical comparison between survivors and nonsurvivors for SAPS II score and its predicted mortality.

Variable	Survivors (*n* = 17) Median (IQR)	Nonsurvivors (*n* = 25) Median (IQR)	MW	*p*
SAPS II	2.9 (1.3–5.8)	53 (37–61)	5.45	< 0.001^∗∗^
Predicted mortality (%)	16 (12–21)	53 (46–57)	5.45	< 0.001^∗∗^

*Note:* IQR: interquartile range, MW: Mann–Whitney test.

Abbreviation: SD, standard deviation.

^∗∗^Highly significant (*p* < 0.001).

### 3.6. Correlational Analysis

**FIGURE 6:**
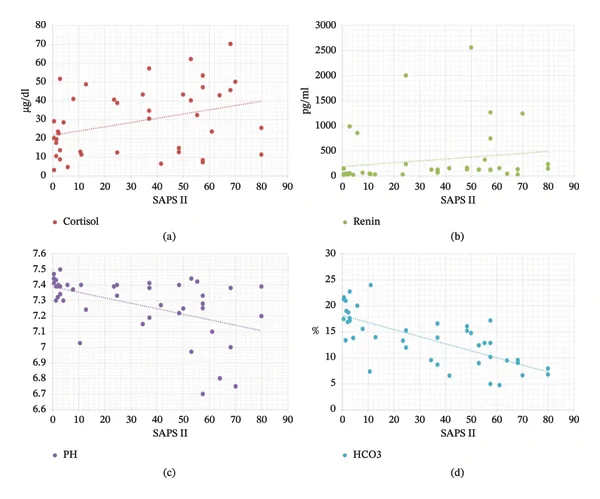
Positive correlation between SAPS II score and cortisol and renin (a and b) and negative correlation between SAPS II score and PH and bicarbonate level (c and d) among the studied cases.

A statistically positive correlation was detected between the SAPSII score and renin and cortisol levels (*p* = 0.001 and 0.03, respectively). In contrast, a statistically negative correlation was detected between the SAPSII score and PH, HCO_3_, and BE values (*p* < 0.001) (Figure 6 a–d).

### 3.7. Validity and Accuracy of Tested Parameters With ROC Curves

**FIGURE 7:**
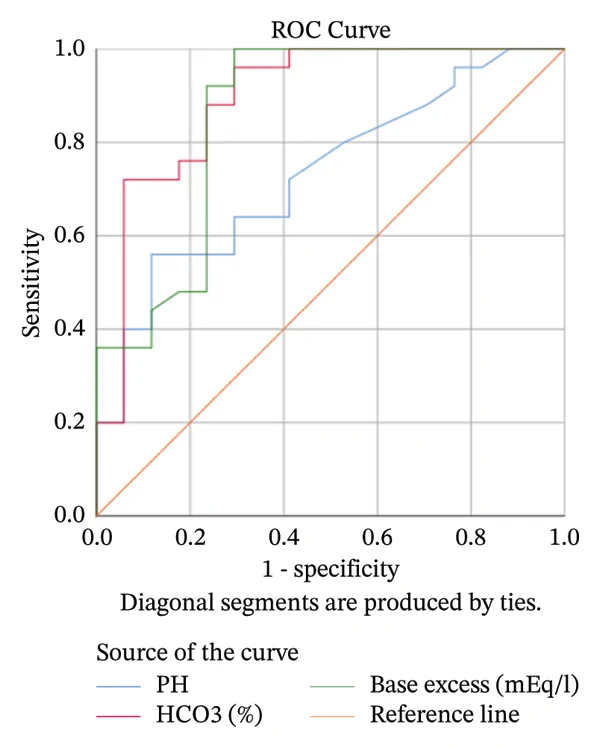
Receiver operator characteristic (ROC) curve showing good validity of bicarbonate and base excess in comparison to the fair accuracy of PH as AlP poisoning prognostic parameters.

**FIGURE 8:**
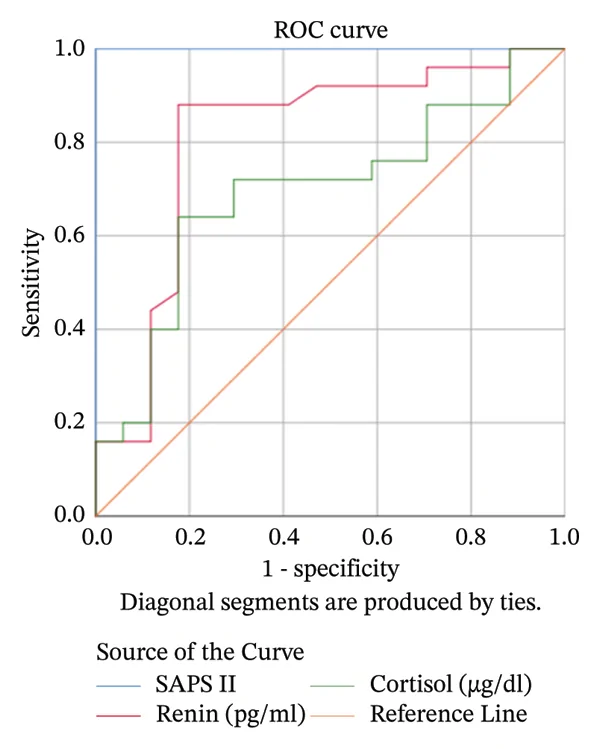
Receiver operating characteristic (ROC) curve showing excellent validity of SAPS II score in comparison with the good predictive accuracy of renin and the poor one of cortisol for AlP studied cases.

Using ROC curves, we identified the most accurate prognostic markers among the studied parameters for predicting mortality in the studied cases by estimating the AUC where the most reliable markers are SAPSII (AUC = 1, excellent), followed by HCO_3_, BE, and renin (AUC = 0.89, 0.86, and 0.81, respectively) of good accuracy (Figures 7 and 8). Table 5 also shows the cut‐off values, specificity, and sensitivity of ABG components, troponin I, cortisol, renin, and SAPS II and reflects troponin I as the least reliable prognostic parameter.

**TABLE 5:** Validity of SAPS II score and laboratory findings in the prediction of mortality among the studied cases using the area under the ROC curve.

Parameter	Cutoff	AUC (95%CI)	*p*	Sen.	Speci.	PPV	NPV	Acc.
PH	< 7.34	0.73 (0.58–0.88)	0.01^∗^	64	70.6	76.2	52.4	64.3
HCO_3_ (%)	< 16.75	0.89 (0.79–1)	< 0.001^∗∗^	96	70.6	82.8	92.3	85.7
Base excess (mEq/L)	< −9.15	0.86 (0.73–0.99)	< 0.001^∗∗^	92	76.5	85.2	86.7	85.7
Troponin (ng/mL)	> 0.1	0.48 (0.31–0.67)	0.90 NS	56	41.2	58.3	38.9	50
Cortisol (μg/dL)	> 23.62	0.69 (0.53–0.85)	0.04^∗^	72	70.6	78.3	63.2	71.4
Renin (pg/mL)	> 66.5	0.81 (0.66–0.96)	0.001^∗^	88	82.4	88	82.4	85.7
SAPS II	> 18.15	1 (1‐1)	< 0.001^∗∗^	100	100	100	100	100

*Note:* Acc.: accuracy, ALT: alanine transaminase, AST: aspartate aminotransferase, CAT: catalase, HCO_3_: bicarbonate, K+: potassium, MDA: malondialdehyde, mEq: milliequivalent, Na+: sodium, NPV:‐ve predicted value, NS: nonsignificant (*p* > 0.05), PH: the potential of hydrogen, PPV: +ve predicted value, SAPS II: simplified acute physiology score, Sen.: sensitivity, SOD: superoxide dismutase, Spec.: specificity.

Abbreviations: AUC, area under the curve; BUN, blood urea nitrogen; CI, confidence interval; TLC, total leukocytic count.

^∗^Significant (*p* < 0.05).

^∗∗^Highly significant (*p* < 0.001).

## 4. Discussion

Pesticide use in developing countries is necessary to improve crop quality and quantity, but the misuse of these chemicals causes severe acute and chronic poisoning [5]. AIP is a widely used pesticide and fumigant in Egypt with high toxicity and mortality rates [13]. The frequency of absolute AlP poisoning from 2017 to 2021 in the East Delta of Egypt was about 5%, which is considerable for a single‐agent poisoning [17]. Death frequently occurs within the first 24–48 h of ingestion, highlighting the need for early identification of patients at high risk of deterioration [2, 5, 11]. This work aimed to evaluate the prognostic value of simple bedside tools and early stress serum markers for predicting the outcome of cases of acute AlP poisoning. The most reliable tested parameters were ABG, serum renin, ECG, and SAPS II score, while a single early measurement of troponin I failed to show a considerable prognostic value. However, the relatively small sample size of 42 poisoned cases who fulfilled the inclusion criteria during the relatively short duration of the study is considered a major statistical limitation to the generalizability of the findings.

Enrolled cases were between 15 and 45 years old, with a mean age of 22.74 ± 8.94. This age range was chosen to avoid any geriatric or pediatric laboratory variations from the adult group values; also, the SAPS II score is applied only for ICU populations over the age of 15 years [16, 18].

Additionally, old age (above 50 years) could elevate SAPS II values in an irrelevant way from the true toxicity manifestations [18]. A similar prevalent age group was reported by previous studies in Egypt [19–21] and other countries [22], where this age group is more susceptible to occupational or intentional exposure.

Most poisoned cases in the current study were females (69%), which is consistent with similar studies [7, 21]. Young females, especially in rural areas, face more stressful conditions such as marital conflict, economic factors, family troubles, blackmail, and educational troubles [13]. On the contrary, Abdel‐Hady et al. [19] reported male prevalence, which could be due to occupational availability [17]. Neither age nor sex showed significant differences between survivors and nonsurvivors, which is similar to the results of Kalawat et al. [23] and unlike El‐Sarnagawy [13], who reported a statistically significant difference in age between survivors and nonsurvivors.

Our results revealed a mortality rate in 59.5%, similar to that reported by Khalifa et al. [6], and Masoud and Barghash [7]. A higher mortality of 70%–90% in severe AlP‐poisoned patients was reported before in several studies [9, 11, 24], while lower mortality was also reported by others [13, 21]. The reported relatively low mortality can be attributed to numerous factors. First, the modest sample size may have influenced mortality estimation and limited generalizability, as small cohorts are more vulnerable to random variation and may not fully represent the full spectrum of disease severity. Second, patients in this study were managed according to a structured institutional protocol at the PCC‐ZUH, which emphasizes early stabilization and aggressive supportive care. All patients were admitted for close monitoring, and most were transferred early to intensive care when indicated. Management included a strict nil per os (NPO) status and early gastrointestinal decontamination using almond, coconut, or paraffin oil (200 mL every 2 h until the onset of diarrhea) to coat the tablet surface and reduce phosphine gas liberation within the gastrointestinal tract [1, 20, 22].

Hemodynamic instability was treated promptly with vasoactive agents and inotropes such as noradrenaline, adrenaline, and dobutamine when required, and suggested supportive therapies have also been adopted, e.g., sodium bicarbonate [25, 26], steroids, N‐acetylcysteine [2], magnesium sulfate, and calcium gluconate [5, 19, 27, 28].

The estimated amount ingested was about one tablet, and it is the common dose reported in different studies [7, 21]. We found that the amount ingested was not significantly related to patient outcome due to several reasons, such as the ingestion of exposed tablets that released their phosphine content, a changed formulation of tablets to granulated powder by crushing and/or dissolving the tablet into the water before ingestion, or vomiting immediately after the ingestion, and this insignificant relation to mortality was reported by previous studies [7, 20, 21]. On the other hand, in other studies, the amount ingested was significantly higher in deceased patients [13, 29, 30].

The time lapse between ingestion and hospital presentation (around 3 hours) was insignificantly different among survivors and nonsurvivors, which aligns with the results of Sanaei‐Zadeh [12], Shadnia et al. [8], and Bogale et al. [29]. This could be explained by the imprecise data given by the victims or their relatives, who were not sure of the exact circumstances of the poisoning. Another explanation is the lack of actual patient intention to end their lives, thus rapidly seeking medical intervention. In most cases, they just tried to gain the attention and compassion of their families [13]. However, Abdel‐Hady et al. [19], Elgazzar et al. [24], and Navabi et al. [30] found it a significant factor.

ABG parameters (HCO_3_, BE, and PH) showed a significant decline in most of the studied AlP‐poisoned cases and were significantly decreased among nonsurvivors. Numerous studies have reported their association with poor prognosis and mortality [6, 11, 21, 24, 31]. Bicarbonate level was considered one of the best prognostic factors predicting mortality [32]. The PH showed good validity as a mortality detector on the ROC curve, which is in harmony with previous studies [7, 33].

The detected metabolic acidosis, more profound in the nonsurvivors, is regarded as the hallmark of AlP toxicity, where shock and tissue hypoxia result in the accumulation of lactic acid that consumes the body’s reserve of HCO_3_ together with impaired oxidative phosphorylation [3, 11, 34]. Metabolic acidosis increases the risk of mortality due to its depressive effect on myocardial contractility [2]. Jaiswal et al. [25] found no significant difference in base excess or PH between the survivors and nonsurvivors on arrival, but the difference became significant 12 hours later, supporting the dynamic nature of acid–base changes in these patients. Rungta et al. [26] reported that early bicarbonate‐driven resuscitation combined with renal replacement therapy may help reverse cardiogenic shock by correcting severe metabolic acidosis and improving left ventricular ejection fraction, systolic blood pressure, and lactate levels. Together, these findings support the importance of early recognition and correction of severe metabolic acidosis in acute AIP poisoning.

Troponin I is one of the excellent markers for myocardial injury. Nonetheless, despite the known AlP cardiotoxicity, the current results showed no statistical increase in troponin I among the studied cases or a significant impact on the outcome by a single early measurement at admission, which is in line with Nayyar and Nair [35]. Karami‐Mohajeri et al. [36] explained that the normal levels of cardiac markers do not disprove cardiotoxicity due to their delayed rise in the serum, while their elevated levels confirm myocardial damage. The kinetics of troponin elevation in AIP toxicity may differ from those seen in ischemic myocardial injury. Myocardial damage in AlP poisoning may evolve, and peak troponin levels may occur later in the clinical course [34].

It is worth mentioning that troponin I was not a significant marker of patient improvement after using a new treatment modality despite amelioration of AlP toxicity with the improvement of cardiac functions [20]. On the contrary, El‐Ebiary et al. [37], Tiwari et al. [38], and Tiwari et al. [38] found a statistical association between serum cardiac troponin and case severity and mortality in AlP poisoning.

Cortisol is a well‐known stress hormone, released in response to critical illness. Its elevation reflects the body’s attempt to maintain hemodynamic stability. The present study revealed a statistically significant association between the rise of cortisol and case mortality. This is consistent with Shadnia et al. [39], who attributed the rise of cortisol levels to the severe psychological stress among the AlP‐poisoned cases who remain conscious until the end stage with severe pain, and due to shock, which develops in these cases. Khalifa et al. [6] demonstrated significantly higher serum cortisol levels among nonsurvivors at admission and during serial measurements. Marked cellular hypoxia activates the adrenal axis, causing a rise in cortisol and stimulating renin release in the blood as well [7].

AlP‐poisoned cases present a scientific dilemma, as serum cortisol levels rise in the early stage of AlP poisoning and then progressively decline in severe, advanced cases [40].

Accordingly, other AlP poisoning studies have reported a significant decrease in cortisol among nonsurvivors [11] or a rise insufficient to match the shock and stress state of poisoned patients, suggesting that normal or low cortisol levels in acute AlP poisoning are abnormal and caused by phosphine‐induced severe adrenal cortex cytotoxicity [41].

There was a significant relationship between the renin activity and case mortality, and it showed a good prognostic performance on the ROC curve (AUC = 0.81). These results were like those found by Masoud and Barghash [7], who reported excellent prognostic potential of renin on the ROC curve, and Saleh and Makhlof [42], who demonstrated a significant rise in renin among nonsurvivors and suggested it as a dynamic marker of cardiovascular stress. Chugh et al. [43] also noticed, long ago, that renin activity is higher in severe AlP toxicity cases compared with healthy controls and shocked patients other than due to AIP poisoning; also, renin activity was directly related to the amount of consumed AlP and the overall mortality.

Standard 12‐lead ECG evaluation is routinely performed for all AlP‐poisoned patients. Significantly more abnormal ECG findings were found among nonsurvivors, as in the results of previous studies [11, 19, 21, 24, 39, 44, 45], demonstrating that cardiac electrical instability remains one of the earliest and most decisive determinants of mortality in AIP poisoning and emphasizing that ECG abnormalities often precede overt hemodynamic collapse. The detected abnormal ECG findings among nonsurvivors in the present study were ischemic changes, followed by ventricular dysrhythmias, and then heart block. This order was previously reported by Soltaninejad et al. [45] and Shadnia et al. [46]. ST‐segment changes were found to be the commonest ECG abnormality associated with mortality by El‐Ebiary et al. [37], while QT prolongation and wide QRS complexes were the strongest predictors of mortality reported by Hadi et al. [47].

The development of ECG changes in AlP poisoning is attributed to phosphine‐induced inhibition of mitochondrial oxidative phosphorylation within cardiomyocytes, with a reduction of the myocardial cellular metabolism and energy depletion, as occurs in ischemia [2, 7]. Moreover, phosphine‐induced oxidative stress disrupts cardiomyocyte membrane action potentially to be seen by the ECG as ischemic‐like changes in the ST segment and the T wave [11, 19, 38, 48].

Several critical case scoring systems were used to assess the severity of acutely AlP‐poisoned patients, like the sequential organ failure assessment (SOFA) score [21] and the SAPS II [10, 18].

The chosen SAPS II in the present work is a hospital‐mortality score that has been developed for about 30 years for evaluating critically ill patients, regardless of the primary illness [15]. It is superior to other scoring systems like SOFA, especially during the first 24 h of patient admission [17]. Furthermore, SAPS II is a simplified version of the APACHE II; so, it was reported to be more practical and reliable for evaluating AlP poisoning [20, 32, 49]. We used the modified SAPS II score customized to AlP‐poisoned cases [8].

In the present work, the SAPS II score showed a highly significant difference between survivors and nonsurvivors, giving a good prognostic potential for AlP cases. The SAPS II score also showed high accuracy (specificity and sensitivity) with excellent predicted mortality on the ROC curve, similar to what was reported before by several studies [2, 18, 32]. However, Masoud and Barghash [7] reported that SAPS II was a good prognostic tool for AlP patients with low specificity (60%) on the ROC curve. The relatively small sample size in the current study may increase the likelihood of overestimation of predictive performance and potential overfitting. Therefore, external validation in larger multicenter cohorts is necessary before drawing definitive conclusions regarding its predictive accuracy in acute AlP poisoning.

There was a statistically significant positive correlation between SAPS II score and cortisol and renin levels and a statistically significant negative correlation between SAPS II score and PH, HCO_3_, and base excess values (*p* < 0.001). Masoud and Barghash [7] also reported a statistically significant positive correlation between SAPS II score and renin but a poor correlation with H_3_CO_3_ and PH.

In conclusion, AlP is a highly toxic substance with a high mortality rate; the early ECG assessment and continuous monitoring, SAPS II score, followed by HCO_3_, base excess, renin, PH, and cortisol levels, arranged in descending order, could help clinicians stratify patients within the first hours of admission identifying candidates for aggressive supportive therapies, ICU admission, and potentially novel interventions such as extracorporeal membrane oxygenation (ECMO) in resource‐equipped settings, ultimately improving patient survival. The limited utility of early troponin I measurement underscores the need to rethink which biomarkers are truly informative in toxic myocardial dysfunction.

Limitations of this study include its single‐center design and relatively small sample size. Laboratory assays were performed only once within the first 24 h, and serial measurements may provide further insight into the temporal behavior of biomarkers. Larger, multicentred studies on a longer time scale are warranted to provide additional insight into disease temporal trajectory and healthcare resource utilization and to explore integration of the results into prognostic scoring systems.

## Funding

This research did not receive any specific grant or funding support from agencies in the public, commercial, or not‐for‐profit sectors.

## Conflicts of Interest

The authors declare no conflicts of interest.

## Supporting Information

Additional supporting information can be found online in the Supporting Information section.

## Supporting information


**Supporting Information** Filled_STROBE_Checklist_AlP_Prospective_Cohort.

## Data Availability

The data that support the findings of this study are available from the corresponding author upon reasonable request.
