# Supplementary material for: Clinical, Biochemical, and Electrocardiographic Predictors of Mortality in Acute Aluminum Phosphide Poisoning
Source: J Toxicol. 2026 Sep 22;2026:4876498. doi: 10.1155/jt/4876498 (PMC13598442; doi:10.1155/jt/4876498)
Supplement: Supplementary file 1 [file JT-2026-4876498-s001.docx]

**STROBE Statement - Checklist of Items for Reports of Observational Studies**

*Completed for: Electrocardiographic Changes, Troponin I, Cortisol, Renin, ABG, and SAPS II score as Early Prognostic Markers in Acute Aluminum Phosphide Poisoning: A Prospective Study*

| **Section / topic** | **Item no.** | **STROBE recommendation** | **Location in manuscript / response** |
| --- | --- | --- | --- |
| Title and abstract | 1(a) | Indicate the study design with a commonly used term in the title or the abstract. | Page 1, Abstract/Methods: described as a prospective cohort study. Title also indicates a prospective study. |
| Title and abstract | 1(b) | Provide in the abstract an informative and balanced summary of what was done and what was found. | Pages 1-2, Abstract: includes background, objective, methods, results, and conclusion, including mortality rate, ECG findings, biomarker AUCs, and SAPS II findings. |
| Introduction - Background/rationale | 2 | Explain the scientific background and rationale for the investigation being reported. | Pages 3-4, Introduction: describes AlP toxicity, high mortality, cardiovascular involvement, ECG abnormalities, biomarkers, ABG parameters, SAPS II, and the evidence gap. |
| Introduction - Objectives | 3 | State specific objectives, including any prespecified hypotheses. | Page 4, final paragraph of Introduction: states that the study assesses the prognostic performance of ECG findings, SAPS II, ABG, troponin I, cortisol, and renin within the first 24 hours. |
| Methods - Study design | 4 | Present key elements of study design early in the paper. | Page 4, section 2.1: states this was a prospective cohort study. |
| Methods - Setting | 5 | Describe the setting, locations, and relevant dates, including periods of recruitment, exposure, follow-up, and data collection. | Page 4, section 2.1: Poison Control Center-Zagazig University Hospitals, Emergency Department, and ICU; January 2021 to June 2021. Page 6, section 2.3: outcome assessed during first 24 hours of admission. |
| Methods - Participants | 6(a) | Cohort study: Give eligibility criteria, and the sources and methods of selection of participants. Describe methods of follow-up. | Pages 4-5, section 2.1: inclusion and exclusion criteria described; 42 consecutive eligible patients enrolled. Page 6, section 2.3: participants classified as survivors or non-survivors according to outcome during the first 24 hours/in-hospital death. |
| Methods - Participants | 6(b) | Cohort study: For matched studies, give matching criteria and number of exposed and unexposed. | Not applicable: this was not a matched cohort study and there were no exposed/unexposed matched groups. |
| Methods - Variables | 7 | Clearly define all outcomes, exposures, predictors, potential confounders, and effect modifiers. Give diagnostic criteria, if applicable. | Pages 4-7, sections 2.1-2.4: exposure/diagnosis of acute AlP poisoning, outcome categories (survivor/non-survivor), poisoning variables, clinical variables, ABG, ECG, biomarkers, and SAPS II scoring are described. Potential confounders/effect modifiers are not formally prespecified. |
| Methods - Data sources/measurement | 8 | For each variable of interest, give sources of data and details of methods of assessment or measurement. Describe comparability of assessment methods if there is more than one group. | Pages 5-6, section 2.2: history, vital signs, GCS, ABG sampling/analyser, serum biomarker sampling, ECG recording and interpretation criteria. Page 6, section 2.3: SAPS II scoring within first 24 hours. |
| Methods - Bias | 9 | Describe any efforts to address potential sources of bias. | Pages 4-6, sections 2.1-2.2: consecutive eligible enrolment and exclusion of uncertain exposure, co-ingestion, chronic systemic disease, and very early ED deaths. Page 6 notes ECG interpretation was not blinded to clinical status or outcome. Bias is partly addressed, but a more explicit bias paragraph would strengthen reporting. |
| Methods - Study size | 10 | Explain how the study size was arrived at. | Page 4, section 2.1: prior sample size estimate by Faculty Biostatistics/Research Support Unit based on in-hospital mortality; minimum required sample size 36; 42 consecutive eligible patients enrolled. |
| Methods - Quantitative variables | 11 | Explain how quantitative variables were handled in the analyses. If applicable, describe which groupings were chosen and why. | Page 7, section 2.4: normally distributed data expressed as mean +/- SD and compared with independent t-test; non-normally distributed data expressed as median/IQR and compared with Mann-Whitney U test. Page 6, section 2.3: patients grouped as survivors and non-survivors by outcome. |
| Methods - Statistical methods | 12(a) | Describe all statistical methods, including those used to control for confounding. | Page 7, section 2.4: SPSS version 27, t-test, Mann-Whitney U test, Chi-square test, Pearson/Spearman correlation, ROC curve analysis, AUC, cut-offs, sensitivity and specificity. No confounder-adjusted modelling reported. |
| Methods - Statistical methods | 12(b) | Describe any methods used to examine subgroups and interactions. | Page 7, section 2.4 and Results: comparisons between survivors and non-survivors were performed. No interaction analyses reported. |
| Methods - Statistical methods | 12(c) | Explain how missing data were addressed. | No missing Data |
| Methods - Statistical methods | 12(d) | Cohort study: If applicable, explain how loss to follow-up was addressed. | Not applicable/partly inherent to design: this was an acute hospital cohort with outcome assessed during admission/first 24 hours. No loss to follow-up is reported. |
| Methods - Statistical methods | 12(e) | Describe any sensitivity analyses. | No sensitivity analyses were performed. |
| Results - Participants | 13(a) | Report numbers of individuals at each stage of study, e.g. potentially eligible, examined, confirmed eligible, included, completing follow-up, and analysed. | Page 4, section 2.1 and page 7, section 3.1: 42 consecutive eligible patients enrolled/included. Full flow of potentially eligible/excluded/completing follow-up is not fully reported. |
| Results - Participants | 13(b) | Give reasons for non-participation at each stage. | Exclusion criteria are provided on pages 4-5, |
| Results - Participants | 13(c) | Consider use of a flow diagram. | Not included. |
| Results - Descriptive data | 14(a) | Give characteristics of study participants and information on exposures and potential confounders. | Pages 7-8, section 3.1 and Table 1: age, sex, amount ingested, lag time, and mortality. Additional clinical characteristics are described in section 3.2 and incorporated into SAPS II. |
| Results - Descriptive data | 14(b) | Indicate number of participants with missing data for each variable of interest. | Not aplicable: All 42 patients had complete data for each variable |
| Results - Descriptive data | 14(c) | Cohort study: Summarise follow-up time, e.g. average and total amount. | acute in-hospital outcome assessed during first 24 hours and survivors discharged after 48 hours after normalisation (page 6). A formal follow-up time summary is not reported. |
| Results - Outcome data | 15 | Cohort study: Report numbers of outcome events or summary measures over time. | Page 7, section 3.1: overall mortality 59.5% (25 deaths), survivors n=17. Outcome classification also described in Methods. |
| Results - Main results | 16(a) | Give unadjusted estimates and, if applicable, confounder-adjusted estimates and their precision. Make clear which confounders were adjusted for and why. | Pages 7-9, Results and Tables 1-5: unadjusted group comparisons, p values, AUCs, cut-offs, sensitivity and specificity reported. No confounder-adjusted estimates or 95% confidence intervals reported. |
| Results - Main results | 16(b) | Report category boundaries when continuous variables were categorised. | Page 9, section 3.7 and Table 5: ROC cut-off values for selected parameters are reported. SAPS II grouping as survivors/non-survivors is outcome-based. If any additional categorisation was used, boundaries should be stated. |
| Results - Main results | 16(c) | If relevant, consider translating estimates of relative risk into absolute risk for a meaningful time period. | Not reported/not applicable: the analysis focuses on ROC performance and group comparisons, not relative risk estimates. |
| Results - Other analyses | 17 | Report other analyses done, e.g. subgroup analyses, interactions, and sensitivity analyses. | Page 9, section 3.6: correlation analyses between SAPS II and renin, cortisol, pH, HCO3, and base excess. Page 9, section 3.7: ROC analyses. No subgroup, interaction, or sensitivity analyses reported. |
| Discussion - Key results | 18 | Summarise key results with reference to study objectives. | Pages 9-10, start of Discussion: summarises that ABG, renin, ECG, and SAPS II were the most reliable tested parameters, while a single early troponin I measurement had limited prognostic value. |
| Discussion - Limitations | 19 | Discuss limitations of the study, taking into account sources of potential bias or imprecision. Discuss both direction and magnitude of any potential bias. | Pages 10 and 16, Discussion/Limitations: small sample size, single-centre design, short duration, single-time laboratory assays, and risk of overestimation/overfitting for SAPS II are discussed. Direction and magnitude of bias are partly discussed but could be expanded. |
| Discussion - Interpretation | 20 | Give a cautious overall interpretation considering objectives, limitations, multiplicity of analyses, results from similar studies, and other relevant evidence. | Pages 10-16, Discussion: findings are interpreted in relation to previous studies and limitations; includes caution regarding small sample size, overfitting, and need for validation. |
| Discussion - Generalisability | 21 | Discuss the generalisability/external validity of the study results. | Page 16, final limitations paragraph: states that larger multicentred studies over a longer time scale are needed to validate findings. Generalisability is addressed indirectly; adding a clearer external validity sentence would strengthen the manuscript. |
| Other information - Funding | 22 | Give the source of funding and the role of the funders. | This research received no specific grant from any funding agency in the public, commercial, or not-for-profit sectors |
